# Supplementary material for: A Likelihood Approach for Real-Time Calibration of Stochastic Compartmental Epidemic Models
Source: PLoS Comput Biol. 2017 Jan 17;13(1):e1005257. doi: 10.1371/journal.pcbi.1005257 (PMC5240920; doi:10.1371/journal.pcbi.1005257)
Supplement: S1 File — (TAR.GZ) [file pcbi.1005257.s014.tar.gz › HSPH_Online-SI-Revision/EnKF07/n10-mild/n10-mild_EnKF2_table8.pdf]

1	newly 3 weeks spec	60.	9.96	{0., 22.}	87.5333	1.79658	newly 3 weeks cumul	239.	187.52	{14., 365.}	91.5481	0.647916	newly 5 weeks spec	59.	5.5	{0., 9.}	92.678	2.00182	newly 5 weeks cumul	350.	199.61	{17., 450.}	85.9286	0.724141	newly 42 weeks cumul	920.	230.21	{17., 559.}	83.012	1.02168
2	newly 3 weeks spec	35.	174.75	{0., 337.}	476.257	1.27258	newly 3 weeks cumul	125.	1137.41	{599., 1543.}	811.416	0.877131	newly 5 weeks spec	13.	118.94	{0., 311.}	894.923	1.41044	newly 5 weeks cumul	172.	1399.23	{762., 1680.}	715.297	0.822232	newly 47 weeks cumul	189.	1941.9	{901., 3878.}	929.206	0.877137
3	newly 3 weeks spec	49.	141.99	{0., 410.}	318.265	1.63525	newly 3 weeks cumul	214.	1268.01	{175., 2255.}	501.079	0.529086	newly 5 weeks spec	21.	57.34	{0., 106.}	302.857	1.60656	newly 5 weeks cumul	265.	1414.83	{231., 3066.}	441.74	0.475487	newly 50 weeks cumul	281.	1544.54	{272., 3931.}	455.993	0.475696
4	newly 3 weeks spec	42.	79.53	{0., 37.}	217.5	0.886976	newly 3 weeks cumul	187.	438.69	{22., 256.}	217.321	0.445812	newly 5 weeks spec	18.	17.72	{0., 15.}	144.	1.23898	newly 5 weeks cumul	235.	491.83	{23., 262.}	197.757	0.463842	newly 45 weeks cumul	289.	522.88	{23., 277.}	179.682	0.507522
5	newly 3 weeks spec	82.	209.26	{3., 731.}	256.707	0.858647	newly 3 weeks cumul	270.	1592.15	{18., 9005.}	562.389	0.716509	newly 5 weeks spec	96.	77.28	{0., 69.}	136.563	1.35428	newly 5 weeks cumul	451.	1778.56	{19., 9447.}	382.302	0.767504	newly 25 weeks cumul	1188.	1951.17	{19., 9466.}	184.211	0.940846
6	newly 3 weeks spec	58.	24.37	{0., 61.}	105.638	1.93772	newly 3 weeks cumul	223.	1379.77	{21., 6814.}	581.673	0.702704	newly 5 weeks spec	18.	16.1	{0., 42.}	145.111	1.64842	newly 5 weeks cumul	288.	1414.96	{27., 6814.}	455.868	0.672072	newly 51 weeks cumul	298.	1488.73	{33., 6814.}	460.433	0.668024
7	newly 3 weeks spec	72.	62.61	{0., 92.}	67.4028	0.619784	newly 3 weeks cumul	231.	931.59	{83., 1718.}	322.55	0.488869	newly 5 weeks spec	20.	27.	{0., 24.}	134.3	0.707989	newly 5 weeks cumul	292.	997.95	{119., 2880.}	261.216	0.443464	newly 47 weeks cumul	336.	1060.25	{146., 3653.}	234.848	0.411048
8	newly 3 weeks spec	137.	108.72	{41., 201.}	57.2409	0.367097	newly 3 weeks cumul	446.	468.21	{193., 701.}	55.1413	0.254125	newly 5 weeks spec	94.	61.95	{5., 123.}	61.5638	0.541499	newly 5 weeks cumul	661.	609.83	{249., 966.}	51.6293	0.267415	newly 42 weeks cumul	1278.	877.29	{300., 1399.}	52.8333	0.342245
9	newly 3 weeks spec	36.	218.28	{13., 674.}	543.389	0.634698	newly 3 weeks cumul	178.	1844.11	{512., 4608.}	936.017	0.778807	newly 5 weeks spec	6.	44.45	{0., 95.}	705.167	1.06582	newly 5 weeks cumul	197.	1979.44	{555., 4839.}	904.792	0.77501	newly 46 weeks cumul	197.	2053.62	{562., 5106.}	942.447	0.795863
10	newly 3 weeks spec	48.	64.9	{0., 256.}	162.833	1.00633	newly 3 weeks cumul	183.	684.65	{18., 672.}	338.454	0.582012	newly 5 weeks spec	54.	71.07	{0., 374.}	194.352	1.74198	newly 5 weeks cumul	294.	821.22	{21., 1489.}	257.537	0.602008	newly 43 weeks cumul	610.	1349.52	{27., 6158.}	231.079	0.763272
11	newly 3 weeks spec	38.	117.32	{5., 141.}	288.684	0.575313	newly 3 weeks cumul	143.	856.93	{87., 1037.}	525.154	0.548244	newly 5 weeks spec	2.	47.13	{0., 83.}	2320.5	1.05565	newly 5 weeks cumul	158.	976.67	{110., 1037.}	537.361	0.524324	newly 48 weeks cumul	209.	1063.83	{125., 1053.}	434.455	0.482651
12	newly 3 weeks spec	4.	193.07	{13., 860.}	4726.75	1.14979	newly 3 weeks cumul	21.	774.96	{68., 2118.}	3590.29	1.12606	newly 5 weeks spec	0.1	127.28	{4., 456.}	127182.	2.35678	newly 5 weeks cumul	21.	1056.28	{82., 4018.}	4929.9	1.23097	newly 50 weeks cumul	21.	1587.37	{96., 7108.}	7458.9	1.32877
13	newly 3 weeks spec	14.	210.33	{28., 460.}	1414.07	0.889189	newly 3 weeks cumul	103.	868.81	{169., 2836.}	751.563	0.667102	newly 5 weeks spec	15.	115.6	{4., 237.}	699.333	0.681128	newly 5 weeks cumul	131.	1138.99	{224., 3365.}	777.656	0.668557	newly 42 weeks cumul	149.	1494.7	{270., 4171.}	911.248	0.716038
14	newly 3 weeks spec	78.	359.31	{0., 1208.}	461.962	1.28593	newly 3 weeks cumul	298.	2759.93	{268., 9287.}	838.258	0.734961	newly 5 weeks spec	48.	55.89	{0., 133.}	149.396	1.6328	newly 5 weeks cumul	412.	2951.	{298., 9598.}	630.364	0.643713	newly 43 weeks cumul	587.	3006.37	{298., 9613.}	428.124	0.528563
15	newly 3 weeks spec	142.	52.41	{0., 143.}	67.5845	1.85094	newly 3 weeks cumul	509.	998.4	{391., 1699.}	111.21	0.212934	newly 5 weeks spec	97.	26.14	{0., 76.}	74.5773	1.81862	newly 5 weeks cumul	755.	1062.04	{418., 1699.}	78.7815	0.223116	newly 45 weeks cumul	1453.	1121.69	{447., 1699.}	70.4205	0.387083
16	newly 3 weeks spec	54.	143.92	{0., 262.}	205.222	0.692029	newly 3 weeks cumul	234.	1400.89	{329., 3112.}	503.098	0.642428	newly 5 weeks spec	35.	31.18	{0., 83.}	73.9429	0.681792	newly 5 weeks cumul	327.	1499.39	{355., 3309.}	364.339	0.543214	newly 46 weeks cumul	351.	1531.93	{360., 3352.}	342.459	0.526193
17	newly 3 weeks spec	90.	278.54	{77., 306.}	223.111	0.47214	newly 3 weeks cumul	301.	1655.63	{976., 1996.}	462.083	0.725851	newly 5 weeks spec	86.	106.36	{8., 118.}	100.744	0.451986	newly 5 weeks cumul	488.	1926.19	{1094., 2182.}	306.977	0.596117	newly 43 weeks cumul	2262.	2122.35	{1148., 2232.}	50.7555	0.276931
18	newly 3 weeks spec	41.	146.13	{4., 607.}	325.585	0.630003	newly 3 weeks cumul	174.	616.38	{183., 1814.}	266.828	0.449642	newly 5 weeks spec	45.	97.44	{0., 384.}	246.444	1.32366	newly 5 weeks cumul	264.	834.9	{196., 2952.}	238.	0.391278	newly 43 weeks cumul	314.	1193.82	{197., 4766.}	308.72	0.423705
19	newly 3 weeks spec	42.	126.64	{6., 389.}	278.381	0.584027	newly 3 weeks cumul	145.	439.58	{27., 1778.}	265.021	0.518458	newly 5 weeks spec	27.	89.82	{3., 135.}	321.852	0.656796	newly 5 weeks cumul	208.	642.68	{39., 2292.}	272.663	0.514621	newly 46 weeks cumul	266.	943.41	{46., 2602.}	319.199	0.550626
20	newly 3 weeks spec	94.	272.29	{10., 790.}	256.351	0.547966	newly 3 weeks cumul	348.	1974.31	{406., 6848.}	467.934	0.51964	newly 5 weeks spec	48.	59.67	{0., 236.}	157.896	1.24377	newly 5 weeks cumul	453.	2163.93	{410., 7471.}	380.947	0.449105	newly 47 weeks cumul	551.	2218.23	{410., 7546.}	311.722	0.403217
21	newly 3 weeks spec	33.	130.12	{0., 292.}	374.364	1.3789	newly 3 weeks cumul	145.	959.21	{534., 1350.}	566.034	0.775918	newly 5 weeks spec	16.	88.35	{0., 241.}	538.688	1.42397	newly 5 weeks cumul	184.	1151.63	{711., 1434.}	530.168	0.761627	newly 47 weeks cumul	205.	1742.04	{860., 3962.}	753.2	0.843405
22	newly 3 weeks spec	79.	319.25	{6., 2007.}	383.329	0.620913	newly 3 weeks cumul	247.	2093.86	{360., 7680.}	748.842	0.594129	newly 5 weeks spec	60.	83.9	{0., 544.}	190.533	1.55198	newly 5 weeks cumul	392.	2343.58	{372., 9412.}	502.005	0.437658	newly 43 weeks cumul	671.	2424.33	{372., 9920.}	288.711	0.344804
23	newly 3 weeks spec	1.	264.89	{34., 816.}	26389.	1.9885	newly 3 weeks cumul	20.	1117.63	{200., 2527.}	5488.15	1.45134	newly 5 weeks spec	0.1	143.88	{4., 471.}	143782.	2.47354	newly 5 weeks cumul	20.	1456.98	{280., 3814.}	7184.9	1.54119	newly 48 weeks cumul	20.	1919.45	{335., 7217.}	9497.25	1.61756
24	newly 3 weeks spec	31.	58.66	{0., 38.}	237.871	1.79084	newly 3 weeks cumul	126.	320.48	{71., 231.}	197.603	0.226624	newly 5 weeks spec	21.	19.29	{0., 15.}	157.571	1.77905	newly 5 weeks cumul	167.	374.27	{71., 254.}	192.317	0.300706	newly 44 weeks cumul	190.	408.83	{71., 292.}	193.132	0.341222
25	newly 3 weeks spec	26.	137.92	{1., 181.}	494.769	0.621854	newly 3 weeks cumul	124.	1131.77	{302., 2668.}	818.298	0.656541	newly 5 weeks spec	3.	13.3	{0., 14.}	458.667	0.979767	newly 5 weeks cumul	138.	1187.23	{305., 2741.}	765.732	0.622518	newly 51 weeks cumul	140.	1192.72	{305., 2744.}	757.286	0.617523
26	newly 3 weeks spec	39.	84.63	{3., 125.}	208.487	0.625423	newly 3 weeks cumul	98.	642.93	{81., 740.}	565.378	0.701215	newly 5 weeks spec	10.	29.55	{0., 46.}	312.5	1.21845	newly 5 weeks cumul	122.	718.73	{90., 923.}	500.156	0.650421	newly 42 weeks cumul	155.	792.29	{90., 952.}	424.626	0.58531
27	newly 3 weeks spec	6.	219.48	{2., 563.}	3586.33	0.859354	newly 3 weeks cumul	37.	1235.81	{57., 3620.}	3247.38	0.973842	newly 5 weeks spec	1.	48.95	{0., 138.}	4853.	1.01355	newly 5 weeks cumul	40.	1389.43	{60., 4055.}	3381.08	0.979648	newly 44 weeks cumul	46.	1448.98	{60., 4769.}	3058.52	0.942351
28	newly 3 weeks spec	58.	337.7	{14., 1597.}	520.31	0.461458	newly 3 weeks cumul	196.	1249.54	{68., 4674.}	565.663	0.454697	newly 5 weeks spec	40.	141.14	{4., 314.}	314.8	0.513043	newly 5 weeks cumul	279.	1613.35	{79., 8081.}	509.251	0.446843	newly 22 weeks cumul	839.	1858.1	{85., 9721.}	213.24	0.547594
29	newly 3 weeks spec	51.	58.38	{0., 123.}	119.098	1.45115	newly 3 weeks cumul	209.	885.75	{52., 2173.}	349.086	0.446516	newly 5 weeks spec	29.	40.33	{0., 84.}	151.414	1.40654	newly 5 weeks cumul	281.	975.47	{59., 2668.}	275.996	0.429171	newly 45 weeks cumul	388.	1147.64	{60., 2973.}	233.273	0.433794
30	newly 3 weeks spec	44.	315.68	{4., 668.}	687.909	0.627852	newly 3 weeks cumul	129.	851.85	{35., 3847.}	585.	0.495993	newly 5 weeks spec	32.	131.75	{0., 616.}	432.719	1.09785	newly 5 weeks cumul	194.	1204.34	{35., 4855.}	554.021	0.48376	newly 44 weeks cumul	268.	1415.66	{35., 6280.}	476.507	0.510419
31	newly 3 weeks spec	39.	43.96	{0., 57.}	160.308	1.8051	newly 3 weeks cumul	120.	1112.06	{83., 1012.}	838.9	0.669708	newly 5 weeks spec	8.	48.21	{0., 36.}	632.125	1.44105	newly 5 weeks cumul	150.	1209.3	{110., 1935.}	717.48	0.628108	newly 51 weeks cumul	165.	1508.64	{161., 6481.}	822.061	0.643585
32	newly 3 weeks spec	110.	433.62	{8., 2285.}	397.964	0.772206	newly 3 weeks cumul	385.	1657.07	{208., 7982.}	356.865	0.358704	newly 5 weeks spec	87.	108.29	{0., 195.}	169.46	1.28382	newly 5 weeks cumul	596.	1993.98	{216., 8802.}	295.211	0.43757	newly 24 weeks cumul	1460.	2124.12	{221., 8950.}	158.752	0.63716
33	newly 3 weeks spec	26.	53.17	{0., 147.}	208.5	1.54342	newly 3 weeks cumul	122.	1091.77	{309., 2540.}	796.73	0.717528	newly 5 weeks spec	8.	26.81	{0., 77.}	339.125	1.37211	newly 5 weeks cumul	152.	1155.67	{355., 2540.}	662.178	0.674636	newly 45 weeks cumul	158.	1219.57	{387., 2540.}	673.753	0.697327
34	newly 3 weeks spec	95.	28.94	{0., 97.}	73.4737	1.93889	newly 3 weeks cumul	318.	826.44	{199., 1818.}	173.308	0.268074	newly 5 weeks spec	73.	15.66	{0., 53.}	79.9726	1.9232	newly 5 weeks cumul	481.	862.95	{252., 1818.}	108.971	0.22059	newly 45 weeks cumul	672.	905.08	{301., 1818.}	90.4881	0.2769
35	newly 3 weeks spec	44.	74.18	{0., 109.}	182.273	1.2463	newly 3 weeks cumul	154.	1113.5	{47., 3984.}	661.558	0.62013	newly 5 weeks spec	28.	35.	{0., 47.}	148.143	1.24959	newly 5 weeks cumul	217.	1198.82	{56., 5563.}	495.493	0.579973	newly 45 weeks cumul	280.	1262.24	{60., 5719.}	396.657	0.543376
36	newly 3 weeks spec	9.	60.51	{0., 116.}	610.333	0.880023	newly 3 weeks cumul	71.	952.89	{133., 871.}	1249.54	0.642775	newly 5 weeks spec	1.	47.47	{0., 57.}	4711.	1.21588	newly 5 weeks cumul	72.	1048.75	{152., 2196.}	1363.01	0.721335	newly 47 weeks cumul	75.	1627.16	{159., 7167.}	2075.25	0.850704
37	newly 3 weeks spec	26.	73.89	{1., 144.}	259.038	0.66846	newly 3 weeks cumul	60.	2059.6	{108., 6981.}	3338.1	1.11239	newly 5 weeks spec	12.	3.69	{0., 14.}	88.9167	1.47585	newly 5 weeks cumul	86.	2079.31	{109., 6985.}	2326.1	0.987286	newly 50 weeks cumul	182.	2081.03	{109., 6985.}	1060.72	0.741854
38	newly 3 weeks spec	105.	214.35	{0., 901.}	231.99	1.84992	newly 3 weeks cumul	339.	1069.41	{99., 3049.}	237.519	0.412413	newly 5 weeks spec	72.	153.06	{0., 626.}	248.083	1.85128	newly 5 weeks cumul	490.	1406.16	{135., 4429.}	216.873	0.38198	newly 46 weeks cumul	831.	2177.59	{205., 6947.}	217.294	0.441454
39	newly 3 weeks spec	5.	184.4	{0., 472.}	3610.4	1.21449	newly 3 weeks cumul	20.	990.14	{236., 1187.}	4852.5	1.31887	newly 5 weeks spec	0.1	118.52	{0., 403.}	118444.	2.15899	newly 5 weeks cumul	20.	1260.07	{240., 2440.}	6202.15	1.40763	newly 47 weeks cumul	20.	1515.82	{240., 5073.}	7480.9	1.46179
40	newly 3 weeks spec	69.	80.29	{2., 264.}	133.783	0.731354	newly 3 weeks cumul	225.	805.98	{74., 2891.}	276.756	0.403544	newly 5 weeks spec	51.	16.06	{0., 28.}	97.7255	1.64181	newly 5 weeks cumul	346.	857.51	{77., 3148.}	174.24	0.306734	newly 20 weeks cumul	809.	875.66	{77., 3157.}	112.232	0.52493
41	newly 3 weeks spec	0.1	53.97	{0., 100.}	53992.	1.11028	newly 3 weeks cumul	0.1	1285.02	{149., 2465.}	1.28492×10^{6}	3.77964	newly 5 weeks spec	0.1	46.55	{0., 51.}	46572.	1.02342	newly 5 weeks cumul	0.1	1383.03	{213., 4655.}	1.38293×10^{6}	3.83734	newly 51 weeks cumul	0.1	1580.06	{285., 7036.}	1.57996×10^{6}	3.90136
42	newly 3 weeks spec	35.	138.53	{13., 124.}	327.8	0.44283	newly 3 weeks cumul	180.	497.43	{62., 506.}	208.917	0.418594	newly 5 weeks spec	44.	81.82	{2., 104.}	169.864	0.629989	newly 5 weeks cumul	253.	685.14	{81., 701.}	201.447	0.38811	newly 43 weeks cumul	319.	1130.38	{115., 1878.}	278.871	0.437646
43	newly 3 weeks spec	0.1	75.88	{0., 113.}	75870.	1.57958	newly 3 weeks cumul	1.	699.8	{356., 691.}	69880.	2.65878	newly 5 weeks spec	0.1	27.29	{0., 58.}	27282.	1.33944	newly 5 weeks cumul	1.	771.67	{399., 729.}	77067.	2.7054	newly 49 weeks cumul	1.	817.82	{412., 875.}	81682.	2.73619
44	newly 3 weeks spec	90.	131.96	{2., 295.}	148.111	0.719791	newly 3 weeks cumul	310.	602.41	{92., 1053.}	161.106	0.373787	newly 5 weeks spec	69.	92.75	{0., 123.}	161.725	1.11979	newly 5 weeks cumul	468.	804.81	{92., 1913.}	156.763	0.457106	newly 45 weeks cumul	766.	1232.75	{92., 3790.}	165.672	0.581188
45	newly 3 weeks spec	7.	113.	{4., 151.}	1530.29	0.959209	newly 3 weeks cumul	18.	1441.78	{199., 951.}	7909.89	1.49787	newly 5 weeks spec	3.	25.32	{0., 75.}	798.	0.997344	newly 5 weeks cumul	26.	1516.71	{200., 985.}	5733.5	1.38324	newly 52 weeks cumul	26.	1561.76	{200., 998.}	5906.77	1.40988
46	newly 3 weeks spec	14.	80.69	{0., 70.}	531.643	0.803215	newly 3 weeks cumul	72.	1075.19	{130., 3365.}	1397.15	0.713592	newly 5 weeks spec	4.	17.29	{0., 22.}	427.25	0.968002	newly 5 weeks cumul	77.	1129.01	{139., 3380.}	1370.35	0.724798	newly 49 weeks cumul	79.	1181.94	{141., 3855.}	1400.33	0.737729
47	newly 3 weeks spec	62.	71.51	{1., 105.}	159.113	1.04699	newly 3 weeks cumul	217.	1127.01	{244., 3819.}	425.24	0.476338	newly 5 weeks spec	38.	22.51	{0., 9.}	136.132	2.06616	newly 5 weeks cumul	302.	1189.13	{257., 3819.}	303.977	0.377483	newly 49 weeks cumul	355.	1212.94	{257., 3849.}	257.73	0.345088
48	newly 3 weeks spec	10.	189.25	{2., 694.}	1826.3	1.04952	newly 3 weeks cumul	99.	990.43	{137., 3041.}	902.515	0.712457	newly 5 weeks spec	18.	62.88	{0., 164.}	318.	1.0192	newly 5 weeks cumul	143.	1162.85	{137., 3406.}	718.329	0.637263	newly 46 weeks cumul	178.	1285.81	{137., 3463.}	632.253	0.612064
49	newly 3 weeks spec	135.	107.68	{7., 338.}	96.637	0.695244	newly 3 weeks cumul	399.	1593.4	{518., 5847.}	302.296	0.404084	newly 5 weeks spec	100.	11.45	{0., 23.}	89.45	1.82923	newly 5 weeks cumul	612.	1638.27	{520., 5991.}	178.819	0.272268	newly 42 weeks cumul	1281.	1647.16	{520., 6001.}	100.512	0.348122
50	newly 3 weeks spec	34.	101.	{9., 216.}	230.059	0.54272	newly 3 weeks cumul	108.	2503.27	{124., 7939.}	2225.36	1.09597	newly 5 weeks spec	11.	12.93	{0., 35.}	139.727	1.00054	newly 5 weeks cumul	144.	2547.33	{147., 7948.}	1678.85	1.00871	newly 42 weeks cumul	204.	2572.89	{158., 7948.}	1174.26	0.900786
